# Knowledge, attitude, practice, needs, and implementation status of intensive care unit staff toward continuous renal replacement therapy: a survey of 66 hospitals in central and South China

**DOI:** 10.1186/s12912-024-01953-6

**Published:** 2024-04-26

**Authors:** Xiaoyan Yu, Lin Ouyang, Jinxiu Li, Ying Peng, Dingming Zhong, Huan Yang, Yanyan Zhou

**Affiliations:** 1 Department of Critical Care Medicine, The Second Xiangya Hospital, Central South University, Changsha, Hunan China; 2 Blood Purification Center, The First Affiliated Hospital of Hunan Normal University, Changsha, Hunan China; 3 Hunan Provincial Center for Critical Care Medicine and Clinical Research in Smart Healthcare, Changsha, Hunan China

**Keywords:** Knowledge, Attitude, Practice, Continuous renal replacement therapy, Intensive care unit staff, Professional education

## Abstract

**Background:**

Continuous renal replacement therapy (CRRT) is a commonly utilized form of renal replacement therapy (RRT) in the intensive care unit (ICU). A specialized CRRT team (SCT, composed of physicians and nurses) engage playing pivotal roles in administering CRRT, but there is paucity of evidence-based research on joint training and management strategies. This study armed to evaluate the knowledge, attitude, and practice (KAP) of ICU staff toward CRRT, and to identify education pathways, needs, and the current status of CRRT implementation.

**Methods:**

This study was performed from February 6 to March 20, 2023. A self-made structured questionnaire was used for data collection. Descriptive statistics, T-tests, Analysis of variance (ANOVA), multiple linear regression, and Pearson correlation coefficient tests (*α* = 0.05) were employed.

**Results:**

A total of 405 ICU staff from 66 hospitals in Central and South China participated in this study, yielding 395 valid questionnaires. The mean knowledge score was 51.46 ± 5.96 (61.8% scored highly). The mean attitude score was 58.71 ± 2.19 (73.9% scored highly). The mean practice score was 18.15 ± 0.98 (85.1% scored highly). Multiple linear regression analysis indicated that gender, age, years of CRRT practice, ICU category, and CRRT specialist panel membership independently affected the knowledge score; Educational level, years of CRRT practice, and CRRT specialist panel membership independently affected the attitude score; Education level and teaching hospital employment independently affected the practice score. The most effective method for ICU staff to undergo training and daily work experience is within the department.

**Conclusion:**

ICU staff exhibit good knowledge, a positive attitude and appropriately practiced CRRT. Extended CRRT practice time in CRRT, further training in a general ICU or teaching hospital, joining a CRRT specialist panel, and upgraded education can improve CRRT professional level. Considering the convenience of training programs will enhance ICU staff participation. Training should focus on basic CRRT principles, liquid management, and alarm handling.

**Supplementary Information:**

The online version contains supplementary material available at 10.1186/s12912-024-01953-6.

## Background

Continuous renal replacement therapy (CRRT) is a widely utilized method of renal replacement therapy (RRT) within intensive care unit (ICU) due to its ability to facilitate precise volume control, stabilize acid-base and electrolyte levels, and maintain hemodynamic stability [1]. CRRT serves as the initial choice of RRT for 75% of critically ill patients [2, 3]. Beyond renal replacement, CRRT finds extensive application across various critical clinical scenarios, including sepsis, poisoning, rhabdomyolysis, volume management, burns, multiple injuries, organ failure, and heatstroke [1, 4–9]. Notably, CRRT has garnered attention amidst the Coronavirus Disease 2019 (COVID-19) pandemic, further underscoring its versatile utility within the ICU setting [10–14]. Consequently, CRRT exhibits a promising scope of application within the ICU [15].

Real-world studies have shown significant variation in the quality of CRRT [16, 17]. The quality of CRRT is closely linked to the level of expertise possessed by the ICU staff [18, 19]. For example, inadequate anticoagulation can result in clotting, leading to unplanned treatment interruptions, while excessive anticoagulation may lead to anticoagulant toxicity or bleeding events [20]. Failing to implement the anticipated CRRT plan can result in wasted medical resources and increased costs. Untreated disturbances in internal environmental balance, such as hyperkalemia and acidosis, can precipitate cardiac arrest [21]. Failure to achieve targeted fluid removal contributes to fluid overload and may compromise patient outcomes requiring CRRT [22].

In the ICU, a specialized CRRT team (SCT) composed of physicians and nurses, playing pivotal roles in administering CRRT [21]. Physicians are tasked with evaluating patients’ eligibility for CRRT, formulating comprehensive treatment protocols, and establishing vascular access, while nurses undertake the actual execution of CRRT. During CRRT, physicians and nurses must jointly monitor the patient’s condition and the equipment, adjusting treatment as needed to ensure optimal outcomes. This inter-professional collaboration is instrumental in the successful deployment of CRRT, significantly enhancing patient outcomes and safety [21, 22].

Numerous studies have shown that the quality of CRRT can be effectively improved by mastering all CRRT-related knowledge, ensuring its correct management, and standardizing its implementation [21–23]. People have been trying to improve quality through various educational training programs [23, 24]. However, there is a paucity of evidence-based research on joint training and management strategies. When available, they are often one-sided surveys of doctors or nurses, with little focus on teamwork and cognitive unity [25]. To bridge this gap, we have developed a Knowledge, Attitude, and Practice (KAP) questionnaire tailored to CRRT. The aim is to thoroughly evaluate the shared knowledge and operational competencies of ICU physicians and nurses throughout the CRRT management continuum. Additionally, it seeks to pinpoint educational needs, preferences, and the current state of CRRT application. These findings will inform the creation of more specialized CRRT educational and training programs.

## Materials and methods

### Study design and setting

This study conducted a cross-sectional survey of ICU physicians and nurses in China. The questionnaire was distributed to ICU medical staff at a number of hospitals in China. The Initial data collection period 1.5 months, from February 6 to March 20, 2023).

### Participants

Study participants comprised conveniently selected ICU medical staff from Central and South China. Each of the seven provinces in Central and South China designated one liaison officer, responsible for their respective liaison tasks. These liaison officers communicated with qualified ICU staff within their province via phone and WeChat to elucidate the study’s objectives.. Upon obtaining consent, a link to the questionnaire was sent for survey completion. Subjects received timely guidance if they encountered any issues while completing the questionnaire. Initially, the questionnaire was disseminated through WeChat, with participants encouraged to share the link with other ICU medical staff. In cases where an insufficient number of responses were received, reminders were sent via voicemail, video, or phone calls. To ensure participant data confidentiality and anonymity, researchers assigned numerical codes instead of names. At the outset of the survey, participants were informed that their completion of the questionnaire implied “consent to participate”.

It is generally accepted that the survey sample size should be at least 5–10 times the number of variables included in a multiple linear regression [26]. Drawing from relevant regulations and both domestic and foreign literature, this study identified 15 variables that likely to influence the KAP of CRRT in the ICU. Considering a 20% attrition rate, a minimum of 180 respondents were deemed necessary.

Inclusion criteria: (1) Registered physicians and nurses in the ICU; (2) Individuals with at least 1 year of experience working in the ICU; (3) Participants who provided signed informed consent (included in the questionnaire). Exclusion criteria: (1) Individuals on leave during the investigation; (2) Regular training students.

### Survey

ICU medical staff were invited to participate in an anonymous survey about CRRT. The researchers utilized a self-made structured questionnaire, with the variable assignments included (see Supplementary File 1). The questionnaire comprised two sections: general information and knowledge, attitude, practice.

#### General information questionnaire

The demographics and institutional information included gender, age, educational level, professional title, hospital grade, teaching hospital or not, years of CRRT practice, administrative role, ICU category, and CRRT specialist panel membership or not. Professional titles were classified as junior, intermediate, and deputy senior or above. Hospital grades were divided into Tertiary Grade A general, secondary specialized, secondary or other [27] (Table 1).

**Table 1:** Demographics and work characteristics of ICU staff (*n* = 395)

Characteristics	
Gender (n, %)	
Male	172 (43.6)
Female	223 (56.4)
Age, years (n, %)	
20–30	94 (23.8)
31–40	201 (50.9)
≥ 40	100 (25.3)
Regional distribution	
Central China	196(49.6)
South China	199(50.4)
Educational level (n, %)	
College or below	40 (10.1)
Bachelor	269 (68.1)
Master or above	86 (21.8)
Professional title (n, %)	
Junior	93 (23.5)
Intermediate	192 (48.6)
Deputy senior or above	110 (27.9)
Hospital grade (n, %)	
Tertiary Grade A general	295 (74.7)
Secondary specialized	11 (2.8)
Secondary or other	89 (22.5)
Teaching hospital (n, %)	
Yes	217 (54.94)
No	178 (45.06)
Working years (n, %)	
1–2	32 (8.1)
3–5	35 (8.9)
6–10	109 (27.6)
≥ 10	219 (55.4)
Years of CRRT practice (n, %)	
<1	80 (20.3)
1–2	69 (17.5)
3–5	110 (27.8)
6–10	84 (21.3)
>10	52 (13.2)
Professional position (n, %)	
Doctor	205 (51.9)
Nurse	190 (48.1)
Administrative personnel (n, %)	
Yes	61 (15.4)
No	334 (84.6)
ICU category (n, %)	
General	352 (89.11)
Specialized	43 (10.89)
CRRT specialist panel membership (n, %)	
Yes	175 (44.3)
No	220 (55.7)
Nurse-patient ratio in CRRT treatment (n, %)	
1:1	182 (46.08)
1: ≥2	213 (53.92)

*ICU* intensive care unit, *CRRT* continuous renal replacement therapy

#### The knowledge, attitudes, and practices questionnaire

The knowledge component was scored using a 5-level Likert scale, with a score of “1” indicating very unfamiliar and “5” indicated very familiar. This section comprised 14 items, allowing for a maximum score of 70 points. A higher score reflected a higher self-rated knowledge level. Similarly, the attitude component was evaluated using a 5-level Likert scale, with “1” indicating highly disagree, and “5” indicating highly agree. With a total of 15 items, this section permitted a maximum score of 75 points, with higher scores indicating a more positive attitude. Regarding the practice component, respondents were presented with two options: “no” and “yes,” scored as 1 and 2, respectively. This section comprised 10 items, totaling 20 points, with a higher score indicating a higher self-rated practice level. Prior to data collection, the questionnaire underwent testing and validation.

The questionnaire was tested and validated before data collection. First, 10 clinical experts assessed the content validity of the questionnaire, and the total content validity of the questionnaire was 0.9. Further, the researchers selected 20 ICU medical staff members meeting the inclusion criteria were selected as a sample. Two questionnaire surveys were conducted on them, with a one-week interval between surveys. The correlation coefficient for the test-retest method was calculated, revealing a high stability with a correlation coefficient of 0.87. Meanwhile, Cronbach’s α was used to test the internal consistency, resulting in a total questionnaire Cronbach’s α coefficient of 0.903. For the sub-questionnaires, the Cronbach’s α coefficients were 0.956 for the knowledge component, 0.831 for the attitude component, and 0.751 for the practice component, indicating excellent reliability and validity.

The hierarchical method of mean distribution was used to classify the knowledge component into three levels: low [14–32], moderate [33–51], and high [52–70]; the attitude component into: low [15–35], moderate [36–55], and high [56–75]; and the practice component into: low [10–13], moderate [14–16], and high [17–20] [28, 29].

It is important to keep the survey as concise and straightforward to reduce the burden on ICU medical staff. The survey consisted of 39 questions and typically required 10–15 minutes to complete.

### Ethics

This study was approved by the Institutional Ethics Committee of the Second Xiangya Hospital of Central South University (No. 2022224). We informed participants in the beginning of the questionnaire that completion of the survey considered “consent to participate”.

### Statistical analysis

Prior to data analysis, data were coded in SPSS (IBM SPSS V.25.0, IBM, Armonk, New York, USA). The Kolmogorov-Smirnov test was applied to assess normality, revealing that the dataset pertaining to knowledge, attitude, and behavior exhibited a normal distribution (*p* > 0.05). Categorical variables and items from the questionnaire with Likert scale items were presented as frequencies and percentages. T-tests were used to compare two subgroups. Analysis of variance (ANOVA) was used to compare more than 2 subgroups. Pearson correlation coefficients was used to analyze correlation between knowledge, attitude, and practice. Multiple linear regression analyses were used to analyze the influence of various demographic and occupational factors on KAP. A *p*-value < 0.05 was considered statistically significant.

## Results

### Demographic and professional characteristics of participants

Out of 405 ICU medical staff from 66 general or specialized hospitals who participated in the survey, 395 provided valid responses after excluding 10 incomplete questionnaires, resulting in an effective response rate of 97.53%. Among the respondents, 56.4% were female, with a slight majority of doctors over nurses at a ratio of 1.079. The distribution of respondents was nearly even between Central China and South China, with a ratio of 0.984. A significant majority, 89.9%, held a bachelor’s degree or higher. In terms of employment, 74.7% were affiliated with tertiary general hospitals, and a substantial 82.9% had over 5 years of experience working in an ICU. Regarding CRRT practice, more than three-fifths of the participants had over 3 years of experience, and 44.3% were members of a CRRT specialist panel. Detailed general information and occupational background data are presented in Table 1.

### Knowledge status

The mean knowledge score was 51.46 ± 5.96 (out of 70). Based on this classification, 61.8% (*n* = 244) of respondents had good knowledge and 4.3% (*n* = 17) had poor knowledge (Table 2).

**Table 2:** KAP level and grading of ICU medical staff on CRRT (*n* = 395)

	Full score	Mean score *(* $\overline{x}$ *±s)*		Grading, *n* (%)	
			Low	Moderate	High
Knowledge	70	51.46 ± 5.96	17 (4.3)	134 (33.9)	244 (61.8)
Attitude	75	58.71 ± 2.19	0 (0.0)	103 (26.1)	292 (73.9)
Practice	20	18.15 ± 0.98	14(3.5)	45(11.4)	336(85.1)

*ICU* intensive care unit, *CRRT* continuous renal replacement therapy

**Fig. 1:**
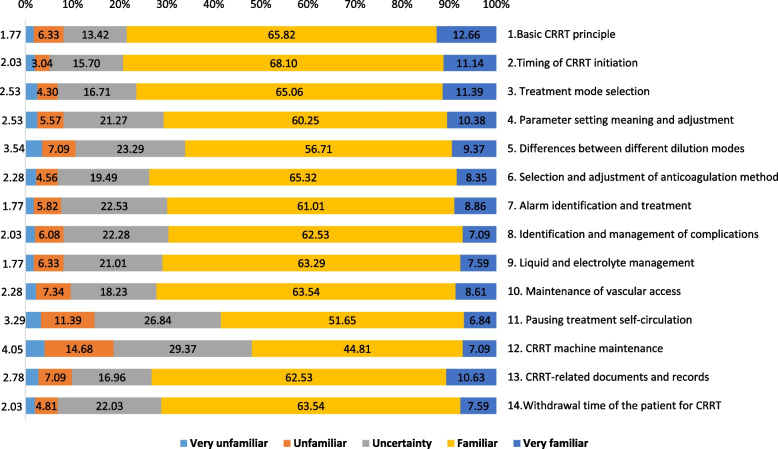
Knowledge of ICU staff toward CRRT. ICU: intensive care unit; CRRT: continuous renal replacement therapy

ICU medical staff knowledge regarding CRRT is presented in Fig. 1. More than 60% of participants were familiar/very familiar with all knowledge items except for CRRT machine maintenance. Basic CRRT principles (65.82% + 12.66%), the timing of CRRT initiation (68.10% + 11.14%), and treatment mode selection (65.09% + 11.39%) were the top three knowledge items. CRRT machine maintenance (4.05% + 14.68%), pausing treatment self-circulation (3.29% + 11.39%), differences between different dilution modes (3.54% + 7.09%) were the top three unfamiliar or very unfamiliar options. Detailed data are presented in Supplementary Table 1.

Table 3 compares the mean knowledge scores of ICU staff based on their demographics and work characteristics. Significant differences were seen in the distribution of knowledge scores between different subgroups, including gender, age, professional title, hospital grade, working years, years of CRRT practice, administrative personnel, ICU category, and CRRT specialist panel membership.

**Table 3:** Comparison of the mean scores of ICU medical staff knowledge, attitude, and practice

Basic information	Mean score*(* $\overline{x}$ *±s)*		
	Knowledge	Attitude	Practice
Gender			
Male	52.85 ± 7.73	59.08 ± 5.63	18.02 ± 2.02
Female	50.37 ± 9.97	58.42 ± 6.20	18.24 ± 1.90
*P*^a^ value	0.007	0.276	0.260
Age, years			
20–30	48.29 ± 10.00	58.18 ± 6.40	18.06 ± 2.14
31–40	52.44 ± 8.01	59.33 ± 5.83	18.08 ± 1.91
≥ 40	52.45 ± 9.80	58.70 ± 5.58	18.36 ± 1.95
*P*^b^ value	0.001	0.014	0.458
Educational level			
College or below	48.85 ± 12.64	56.45 ± 6.87	17.65 ± 2.77
Bachelor	51.62 ± 8.40	58.63 ± 5.94	18.10 ± 1.85
Master or above	52.14 ± 9.36	60.00 ± 5.26	18.55 ± 1.75
*P*^b^ value	0.148	0.007	0.041
Professional title			
Junior	47.91 ± 10.06	57.17 ± 5.82	18.12 ± 2.06
Intermediate	51.59 ± 8.85	58.80 ± 6.13	18.03 ± 2.01
Deputy senior or above	54.21 ± 7.81	59.85 ± 5.54	18.39 ± 1.74
*P*^b^ value	<0.001	0.006	0.291
Hospital grade			
Tertiary Grade A general	52.28 ± 8.43	58.86 ± 5.86	18.30 ± 1.80
Secondary specialized	51.55 ± 10.38	58.73 ± 4.80	18.09 ± 2.17
Secondary or other	48.71 ± 10.69	58.20 ± 6.43	17.65 ± 2.33
*P*^b^ value	0.005	0.66	0.022
Teaching hospital or not			
Yes	58.79 ± 8.80	58.76 ± 6.04	18.37 ± 1.80
No	51.05 ± 9.55	58.65 ± 5.88	17.88 ± 2.10
*P*^a^ value	0.426	0.85	0.014
Working years			
1–2	45.19 ± 9.41	55.81 ± 6.65	18.09 ± 2.62
3–5	47.77 ± 9.08	57.71 ± 4.99	17.91 ± 1.99
6–10	51.59 ± 8.09	58.61 ± 5.60	18.23 ± 1.80
≥ 10	52.90 ± 9.11	59.34 ± 6.06	18.16 ± 1.92
*P*^b^ value	<0.001	0.011	0.870
Years of CRRT practice			
<1	43.06 ± 11.35	56.45 ± 6.05	17.99 ± 2.36
1–2	50.38 ± 7.75	57.99 ± 5.74	17.90 ± 1.95
3–5	52.41 ± 5.62	58.24 ± 5.24	17.96 ± 1.92
6–10	55.81 ± 6.25	61.15 ± 6.09	18.54 ± 1.41
>10	56.75 ± 7.80	60.19 ± 5.80	18.50 ± 1.93
*P*^b^ value	<0.001	<0.001	0.105
Professional position			
Doctor	51.65 ± 8.79	58.87 ± 5.34	18.19 ± 1.94
Nurse	51.24 ± 9.52	58.54 ± 6.58	18.11 ± 1.97
*P*^a^ value	0.655	0.582	0.666
Administrative personnel			
Yes	55.20 ± 7.40	60.08 ± 5.66	18.03 ± 1.80
No	50.77 ± 9.27	58.46 ± 5.99	18.17 ± 1.98
*P*^a^ value	<0.001	0.049	0.613
ICU category			
General	51.84 ± 8.90	58.89 ± 5.86	18.17 ± 1.96
Specialized	48.33 ± 10.53	57.23 ± 6.60	17.98 ± 1.94
*P*^a^ value	0.017	0.085	0.540
CRRT specialist panel membership			
Yes	54.81 ± 6.80	60.34 ± 6.41	18.29 ± 1.90
No	48.80 ± 9.87	57.41 ± 5.24	18.03 ± 1.99
*P*^a^ value	<0.001	<0.001	0.180
Nurse-patient ratio in CRRT treatment			
1:1	50.98 ± 9.35	58.29 ± 6.10	18.12 ± 1.94
1: ≥2	51.86 ± 8.96	59.07 ± 5.83	18.17 ± 1.97
*P*^a^ value	0.343	0.193	0.829

*ICU* intensive care unit, *CRRT* continuous renal replacement therapy

^a^T-test was used for comparison between the two subgroups

^b^ANOVA was used for comparison among more than 2 subgroups

*P* value < 0.05 was considered as a significant difference

### Attitude status

The mean attitude score was 58.71 ± 2.19 (out of 75). The higher the score, the more positive the respondent’s attitude toward CRRT. 73.9% (*n* = 292) of respondents had a positive attitude and no respondent scored below 35 (Table 2).

**Fig. 2:**
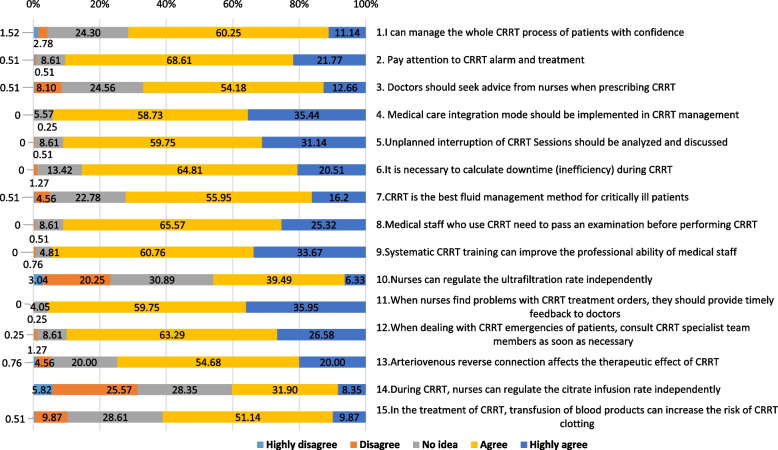
Attitude of ICU staff toward CRRT. ICU: intensive care unit; CRRT: continuous renal replacement therapy

The attitude of ICU medical staff toward CRRT is presented in Fig. 2. More than 90% of participants highly agreed or agreed with these 5 points: (1) The CRRT management in the ICU should adopt a collaborative model involving physicians and nurses. (35.44% + 58.73%); (2) When nurses find problems with CRRT treatment orders, they should provide timely feedback to doctors (35.95% + 59.75%); (3) Systematic CRRT training can improve the professional ability of medical staff (33.67% + 60.76%); (4) Medical staff who use CRRT need to pass an examination before performing CRRT (25.32% + 65.57%); and (5) Pay attention to CRRT alarms and treatment (21.77% + 68.61%). The two most highly disagreed or disagreed views were: (1) During CRRT, nurses can regulate the citrate infusion rate independently (5.82% + 25.57%); and (2) Nurses can regulate the ultrafiltration rate independently (3.04% + 20.25%). Detailed data are presented in Supplementary Table 2.

Table 3 compares the mean attitude scores of ICU staff based on their demographics and work characteristics. There were significant differences in the distribution of attitude scores between different subgroups, including age, educational level, professional title, working years, years of CRRT practice, administrative personnel, and CRRT specialist panel membership.

### Practice status

The mean practice score was 18.15 ± 0.98 (out of 20). The higher the score, the more appropriate the practice. When ranges of 10–13, 14–16 and 17–20 were defined as low, moderate, and high, respectively, 85.1% (*n* = 336) had a high practice score and only 3.5% (*n* = 14) had a low practice score (Table 2).

**Fig. 3:**
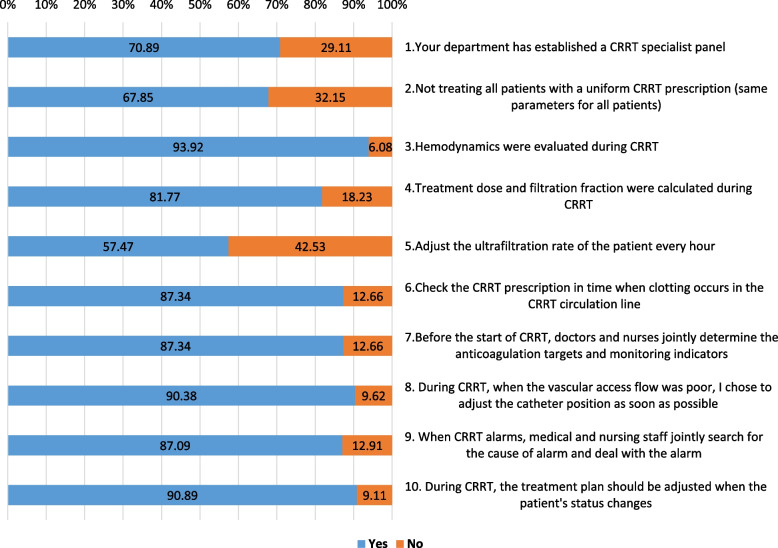
Practice of ICU staff toward CRRT. ICU: intensive care unit; CRRT: continuous renal replacement therapy

The practices of ICU medical staff regarding CRRT are present in Fig. 3. Over 90% of participants indicated they would monitor hemodynamics during CRRT (93.92%) and believed that the CRRT plan should be adjusted at any time according to the patients’ condition (90.89%). During CRRT, when the vascular access flow was poor, 90.38% of respondents would first adjust the position of the catheter. Regarding decision-making, 87.34% of respondents believed that doctors and nurses should make joint decisions on the formulating anticoagulation methods and goals, while 87.09% believed that ICU doctors and nurses should work together to solve alarms. Similarly, when clotting occurred, 87.34% of respondents recognized that CRRT settings may need to be examined and adjusted rather than solely blaming the care process. A CRRT specialist panel was established at 70.89% of the participants’ departments. However, it is concerning that 32.15% of respondents still use a uniform CRRT prescription, only 57.47% regulated the ultrafiltration rate hourly, and 18.23% did not calculate the therapeutic dose and filtration fraction when prescribing. Detailed data are presented in Supplementary Table 3.

Table 3 compares the mean practice scores of ICU staff based on their demographics and work characteristics. There were significant differences in the distribution of practice scores between different subgroups, including educational level, hospital grade, and teaching hospital employment.

### Correlation analysis

Pearson correlation coefficient tests showed a significantly positive correlations between knowledge and attitude (*r* = 0.431, *p* < 0.001), knowledge and practice (*r* = 0.250, *p* < 0.001), and attitude and practice (*r* = 0.176, *p* < 0.001). Detailed data are presented in Supplementary Table 4.

### Multiple linear regression analysis

After multiple linear regression analysis, we found that: Gender, age, years of CRRT practice, ICU category, and CRRT specialist panel membership independently affected knowledge score. Educational level, years of CRRT practice, and CRRT specialist panel membership independently affected attitude score. Education level and teaching hospital employment independently affected practice score. Detailed data are presented in Table 4.

**Table 4:** Multiple linear regression of knowledge, attitude and behavior of ICU medical staff on CRRT

Variable	Knowledge			Attitude			Practice		
	β	SE	*P* value	β	SE	*P* value	β	SE	*P* value
Gender	−0.103	0.835	0.024	–	–	–	–	–	–
Age	−0.203	0.872	0.007	−0.104	0.652	0.231	–	–	–
Educational level	–	–	–	0.152	0.565	0.004	0.409	0.177	0.021
Professional title	0.001	0.958	0.987	−0.021	0.700	0.807	–	–	–
Hospital grade	−0.065	0.531	0.182	–	–	–	−0.170	0.139	0.220
Teaching hospital	–	–	–	–	–	–	− 0.442	0.196	0.025
Working years	0.087	0.681	0.211	0.083	0.499	0.290	–	–	–
Years of CRRT practice	0.437	0.399	<0.001	0.178	0.264	0.002	–	–	–
Administrative personnel	−0.035	1.148	0.446	− 0.014	0.847	0.792	–	–	–
ICU category	−0.091	1.277	0.037	–	–	–	–	–	–
CRRT specialist panel membership	−0.227	0.846	<0.001	−0.209	0.623	<0.001	–	–	–

*ICU* intensive care unit, *CRRT* continuous renal replacement therapy

Knowledge: F = 20.155, R^2^ = 0.304; Attitude: F = 7.374, R^2^ = 0.102; Practice: F = 5.801, R^2^ = 0.032

*P* value < 0.05 was considered as a significant difference

### CRRT educational pathways and needs

**Fig. 4:**
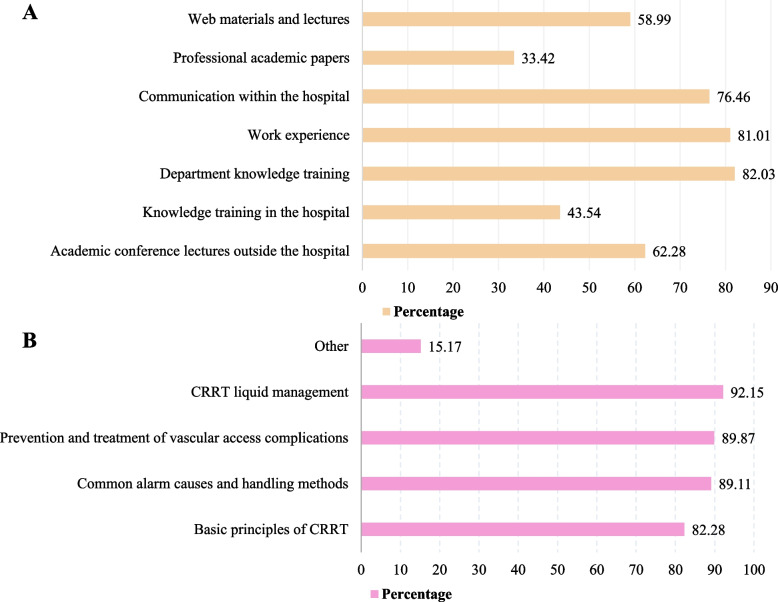
CRRT education pathway and the needs of ICU staff. **A** Education pathway of CRRT, (**B**) Training and learning content needs. ICU: intensive care unit; CRRT: continuous renal replacement therapy

CRRT information was acquired by 82.03% of participants through department knowledge training, 81.01% through work experience, and 76.46% through communication within the hospital. Only 33.42% of respondents reported education through professional papers (Fig. 4A).

Educational needs were CRRT liquid management (92.15%), prevention and treatment of vascular access complications (89.97%), common alarm causes and handling methods (89.11%), and basic CRRT principles (82.28%) (Fig. 4B).

### CRRT implementation status

#### Volume assessment

**Fig. 5:**
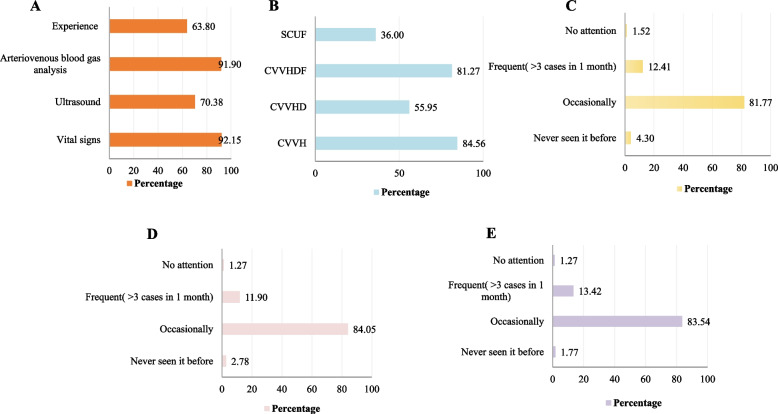
Investigation of CRRT in ICU medical staff. **A** Volume assessment methods, (**B**) Commonly selected CRRT mode, (**C**) Frequency of CRRT circuit clotting, (**D**) Frequency of unplanned interruption, (**E**) Frequency of hypotension in CRRT. ICU: intensive care unit; CRRT: continuous renal replacement therapy; CVVH: continuous venovenous hemofiltration; HP: hemoperfusion; CVVHDF: continuous venovenous hemodiafiltration; SCUF: slow continuous ultrafiltration

Monitoring vital signs remained the primary method for assessing volume during CRRT (92.15%), followed by monitoring arteriovenous blood gas analysis (91.90%). Assessments were more commonly by ultrasound (70.38%) than empirical assessment (63.8%). It should be noted that this section was multiple choice, so the above four methods could have been used in combination (Fig. 5A).

#### Mode selection

Continuous venovenous hemofiltration (CVVH) (84.56%) and continuous venovenous hemodiafiltration (CVVHDF) (81.27%) were the most commonly selected modes, followed by continuous venovenous hemodialysis (CVVHD) (55.95%) and slow continuous ultrafiltration (SCUF) (36%) (Fig. 5B).

#### Circuits clotting, unplanned interruption and hypotension

Occasional circuit clotting during CRRT was reported by 81.77% of the participants while 12.41% had frequent circuit clotting (> 3 cases in 1 month) (Fig. 5C). Similarly, 84.05 had occasional unplanned interruptions during CRRT and 11.9% had frequent unplanned interruptions (> 3 cases in 1 month). (Fig. 5D). Occasional hypotension during CRRT was reported by 83.54 while 13.4% reported frequent hypotension (> 3 cases in 1 month) (Fig. 5E).

## Discussion

CRRT is an important organ support method that has been widely used in the field of critical care medicine [5, 6, 9, 11, 13, 30]. To ensure patients receive precise and high-quality treatment, comprehensive investigations into all aspects of CRRT have been conducted. The timing, mode, therapeutic dose, anticoagulation method, hemodynamic monitoring, and various special modes represent focal points of research [31–37]. The applications of CRRT have expanded beyond traditional ward to various surgeries, particularly organ transplantation procedures [35, 38, 39]. Additionally, CRRT plays a role in temperature control for critically ill patients, such as rewarming individuals experiencing severe accidental hypothermia-systematic [40]. The quality of CRRT can affect patient prognosis [41, 42].

CRRT is a medical procedure necessitating close cooperation between doctors and nurses, and the involvement of highly skilled SCT professionals can enhance its implementation success rates [20–22]. The following six steps had aligned with the theme of promoting high-quality CRRT through precision medicine methods, as proposed by the 17th International Acute Dialysis Quality Initiative (ADQI) [17, 43]. They were: 1) Close collaboration between critical care medicine and nephrology; 2) Define the primary goal of CRRT daily; 3) Keep CRRT running; 4) Review the appropriateness medication dosing daily; 5) Ensure appropriate nutrition support during CRRT; 6) Avoid CRRT-related complications. The lack of KAP studies of CRRT on both physicians and nurses in critical care, especially in terms of care collaboration and knowledge sharing, has prevented us from developing more efficient and targeted measures to improve CRRT quality. To fill this gap, we designed a KAP questionnaire. The design of this KAP survey focuses on steps 2, 3, and 4. In view of the importance of cooperation among SCT members in the implementation of CRRT [21], we focused on understanding the answer choice of physicians and nurses for the same question, so as to provide more basis for the improvement of the integration cooperation.

### The survey population has a well-representation

The high completion rate (97.53%) of this questionnaire indicates that the participants were willing to participate and their careful consideration of each question. Data from Table 1 suggest that our survey results are a reasonably represent the target population. Subgroup analyses and comparisons could be conducted with similar sample sizes to investigate the influence of various factors on knowledge, attitude, and practice.

### ICU staff have good knowledge of CRRT

More than two fifths of participants exhibited good knowledge, with the top three areas being basic CRRT principles, the timing of CRRT, and treatment mode selection. Since the first continuous arteriovenous hemofiltration (CAVH) treatment was performed in 1977 [44], researchers have constantly explored methods to enhance CRRT. The timing of CRRT initiation has long been a research focus [45–49], leading to the development of various CRRT modes, including continuous arteriovenous hemodiafiltration (CAVHDF), continuous arteriovenous hemodialysis (CAVHD), CVVH, CVVHD CVVHDF and integrated technology [50].

The lowest three knowledge scores were observed in CRRT machine maintenance, pausing treatment self-circulation, and differences between different dilution modes.. Medical staff primarily function as users, requiring the assistance of professional engineers for machine-related issues. “Pausing treatment self-circulation” refers to the machine entering a self-circulation state during the CRRT process due to a temporary interruption, resuming treatment when conditions permit [51]. Many hospitals in China lack mobile bedside computed tomography and magnetic resonance imaging, necessitating patients to leave the ICU to complete these examinations. Hence, it’s crucial to fully comprehend pausing treatment self-circulation. Dilution modes are closely related to the therapeutic dose, filtration fraction, and filter life span, even affecting the patency of the entire CRRT circuit [1, 52, 53]. Dilution modes are dynamic and complex processes. A lack of in-depth understanding may be the reason for the poor familiarity with these.

Based on Table 3, it seems that male, age, hospital grade, extended working years and CRRT practice time, administrative personnel, CRRT specialist panel membership, and working at a general ICU were positively correlated with knowledge scores. However, our multiple linear regression analysis found that only male sex, extended CRRT practice time, working at a general ICU, and CRRT specialist panel membership were positively associated with knowledge scores. Surprisingly, there was a negative correlation between age and knowledge scores. There are several explanations for these findings. Firstly, this study relied on subjective surveys, allowing participants to self-assess their knowledge levels. A study on confidence in pediatric endotracheal intubation found that male interns reported higher initial confidence levels compared with females [54]. This confidence wasn’t linked to prior experience in airway management or intubation performance [54]. Similarly, our results showed that males exhibited higher confidence levels in their knowledge compared to females. Secondly, longer CRRT practice duration provides staff with more opportunities to deepen their knowledge. Additionally, CRRT specialist panel members benefit from regular study sessions and increased peer communication. Thirdly, compared to specialized ICUs, general ICUs encounter a broader range of diseases, necessitating staff to possess more comprehensive professional knowledge. Lastly, the observed positive correlation between age and knowledge level (Table 3) initially suggested that older participants, who had longer work experience and CRRT practice, possessed higher knowledge scores. However, upon controlling for these variables using multiple linear regression analysis (Table 4), it became evident that advancing age was associated with lower knowledge scores. This discrepancy could be attributed to the decline in working memory (WM) capacity with age. As individuals age, their WM capacity diminishes, leading to slower reaction times and reduced accuracy [55]. Consequently, the capacity to acquire, process, and retain knowledge, as well as cognitive functions, gradually diminishes [55, 56].

### The attitude of ICU staff toward CRRT were very positive

The mean attitude score of ICU staff was high. It is noteworthy that 73.9% of participants had a positive attitude toward CRRT. Additionally, 90% of ICU staff reported timely handling of CRRT alarms, along with doctor-nurse integration, pre-job assessment, systematic training, and timely feedback adjustment between doctors and nurses.

31.39% of the participants expressed a negative attitude toward ICU nurses independently regulating the citrate infusion rate. Citrate serves not only as an anticoagulant but also plays a role in energy metabolism through the tricarboxylic acid cycle, impacts acid-base balance, and involves a variety complex metabolic sites [57, 58]. The regulation of citrate infusion rate is intricate and cannot rely on a single index for monitoring. Incorrect monitoring regulation may lead to poisoning or inadequate anticoagulation [59], thus contributing to ICU staff’s lack of confidence in this practice. Similarly, 23.29 of the participants disagreed with allowing nurses to independent regulate the ultrafiltration rate. The regulation of the ultrafiltration rate must consider various factors, including the patient’s hemodynamics, fluid balance, and organ function [60]. A net ultrafiltration (NUF) rate > 1.75 mL/kg/h was associated with increased mortality compared with an early NUF rate < 1.01 mL/kg/h [61]. The complexity of the mechanism leads participants to think that it is difficult.

According to Table 3, age, educational level, professional title, extended working years and CRRT practice time, administrative personnel, and membership on a CRRT specialist panel showed positive correlations with attitude scores. However, multiple linear regression analysis identified only three remaining factors: education level, extended CRRT practice time, and joining a CRRT specialist panel. Several reasons account for this observation. Firstly, higher educational attainment and longer CRRT practice duration correlate with increased exposure to CRRT information, thereby fostering a more positive attitude among staff. A study examining parental knowledge and attitudes toward epilepsy similarly found that higher education levels correlated with greater awareness and positivity towards epilepsy [62]. Secondly, belonging to a CRRT specialist panel may enhance medical staff’s perception of CRRT. Research on nurses indicated that a strong professional identity positively influences emotional regulation, social support, and professional success [63]. Additionally, participation in specialist panels often involves setting career goals, which can bolster self-efficacy and enthusiasm, contributing to a more positive attitude [64].

The above results indicated that upgrading educational level, extended CRRT practice time, and joining a CRRT specialist panel could improve the attitude of ICU staff.

### The appropriateness of ICU staff CRRT practice was good

The mean practice score high. Most (85.1%) participants scored high, the level of practice matched knowledge and attitude.

We were pleased to note that the vast majority of medical staff (> 90%) monitor hemodynamics during CRRT and advocate for real-time adjustments based on change in the patient’s condition. Hemodynamic instability related to renal replacement therapy (HIRRT) can increase mortality rates and delay renal function recovery [34]. Understanding the mechanisms of HIRRT and strategies to mitigate its occurrence has been a recent research focus [34, 65, 66]. Medical staff should adjust the ultrafiltration rate, treatment time, dose, dialysate sodium concentration, dialysate calcium concentration, dialysate temperature, and buffer system according to the patient’s condition to reduce the incidence of HIRRT [66].

Despite their practice scores, nearly 1/3 of participants currently use a uniform CRRT prescription (same parameters for all patients). This practice poses risks due to the heterogeneity of critically ill patients. Prioritizing understanding individual patient requirements for renal support, such as electrolyte imbalance, fluid overload, or systemic removal of inflammatory mediators, is crucial before prescribing a tailored treatment plan [67].

Only 57.47% of participants adjusted their ultrafiltration rate hourly, despite over 90% being hemodynamically monitored during treatment. This discrepancy may stem from the heavy workload and inadequate energy levels among ICU staff. ICU staff. Additionally, those adjusting the ultrafiltration rate may not fully grasp the necessity of precise hourly adjustments, indicating a need for further training in this area.

In contrast to knowledge and attitude, the practice scores of CRRT specialist panel members were not higher than those of non-specialist group participants (*p* = 0.180, Table 3). The reasons for this may be as follows: 1) mastering CRRT requires extensive practical experience. Mere membership in a CRRT specialist panel, without significant practical experience, may lead to quicker improvements in knowledge and attitude but does not necessarily enhance practical skills; 2) the absence of a CRRT specialist panel in 29.11% of participant departments may prevent individuals with strong practical abilities from joining such panels. Encouraging the establishment of specialized groups and reinforcing practical training is imperative.

Both educational level and employment in teaching hospitals independently influence practice scores. Previous research suggests that the educational level of nurses plays a crucial role in fostering professional awareness and integrating professional values into practice [68]. Nurses with higher educational levels demonstrate greater proficiency in detecting adverse reactions during treatment [69]. Teaching hospitals are associated with superior quality of care [70], improved postoperative outcomes [71], and reduced in-hospital mortality rates [72]. Additionally, medical staff at teaching hospitals assume teaching responsibilities alongside their clinical duties, leading them to subconsciously ensure practice standardization. Moreover, teaching hospitals implement more rigorous assessment systems, further enhancing practice standardization.

The above results indicate that by upgraded educational level and further study at a teaching hospital can improve the practice quality of ICU staff.

### Good knowledge and a positive attitude may lead to correct practice

Our correlation analysis revealed significant associations among knowledge, attitude, and practice. Correlation coefficients < 0.35 were considered low or weak correlations, 0.36 to 0.67 modest or moderate correlations, and 0.68 to 1.0 strong or high correlations [73]. The correlation between knowledge and attitude was moderately positive (*r* = 0.431). Both knowledge (*r* = 0.250) and attitude (*r* = 0.176) exhibited weak positive correlations with practice, with knowledge demonstrating a stronger correlation than attitude. These results suggest that while possessing a good level of knowledge and a positive attitude is beneficial, they alone may not suffice for ensuring good practice. Implementation of standardized processes, stringent systems, and scientific assessment mechanisms are also imperative for fostering and sustaining good practices.

### Department knowledge training and work experience are the main ways to receive CRRT education and training

The condition of critically ill patients can change rapidly, necessitating uninterrupted monitoring and treatment by ICU staff 24 hours a day [74]. In-department training is the most effective approach, as it allows staff to integrate learning into their daily routines without requiring additional time or a change in location. Consequently, when developing training programs, it is crucial to consider both time and location to enhance participation willingness among ICU staff.

Training sessions should focus on topics of interest to the participants, including CRRT liquid management, prevention and treatment of vascular access complications, common alarm causes and handling methods, and the basic CRRT principles.

### Volume assessment was not precise enough, CVVH and CVVHDF were the mainstream modes

Our study showed that volume assessment primarily relied on monitoring vital signs and arteriovenous blood gas analysis, which are considered macro assessment methods. However, only 70.38% of respondents reported using critical ultrasound for volume assessment during CRRT. Ultrasound offers intuitive, real-time, and accurate measurements, making it widely applicable throughout the ICU. Dynamic monitoring of the inferior vena cava diameter (IVCD) and variability via ultrasound can assess volumetric status, guide dehydration adjustment during CRRT, and expedite relief of heart failure symptoms in patients with renal and acute heart failure [75]. Additionally, Lung ultrasound score assess pulmonary edema in pediatric acute respiratory distress syndrome patients undergoing CRRT [76].. Furthermore, echocardiography can be used to identify pulmonary hypertension and left and right ventricular systolic dysfunction in patients with CRRT [77]. Promoting the use of critical ultrasound in CRRT can enhance the precision of volume assessment and fluid management.

More than 4/5 of participants preferred CVVH and CVVHDF modes. Mode selection varied depending on therapeutic objectives, solute removal, hemodynamic conditions, and the medical staff’s familiarity with CRRT modes. CVVHDF, combining convection and diffusion, emerged as the most prevalent mode, boasting an extended cardiopulmonary bypass life [43, 78].

### Limitations

Firstly, the survey content did not involve interdisciplinary cooperation, nutritional support, or medication regulation, which are integral to the implementation of high-quality CRRT [20]. Secondly, the survey did not inquire about the annual number of CRRT procedures performed by the participating departments, a factor that may better correlate with professional experience than years of CRRT practice. Thirdly, the questionnaire did not involve the combination of artificial intelligence and CRRT. The development of CRRT has been upgraded from a simple technological revolution to the cross-field cooperation between artificial intelligence and CRRT [79]. Suggestions have been made regarding the utilization of chemical sensors for maintaining acid-base balance and electrolytes, facilitating continuous adjustment of dialysate and replacement fluid composition [79]. Additionally, there is potential for the development of miniaturized wearable or implantable devices for monitoring and treating critically ill ICU patients requiring blood purification [80]. Fourthly, the survey lacks comparisons with other countries and regions. Regrettably, there remains a notable dearth of comprehensive KAP surveys pertaining to CRRT. Existing surveys predominantly targeted either physicians, nurses, or patients, neglecting a holistic perspective [25, 81, 82]. This study primarily emphasized the optimization of CRRT quality within the ICU context, rather than delving into patient prognosis, the selection of kidney replacement modalities, or the intricacies of chronic patient self-management. Fifthly, We indeed employed a “convenient” rather than “random” method for selecting ICU staff for this survey. This could lead to bias in the study results, as participants who volunteer for the survey may have a more positive attitude. Finally, the survey did not assess the economic development status of the participants’ regions. Limited resources may cause clinicians to encounter various barriers to CRRT, including a limited number of ICU staff and trained personnel, knowledge gaps, poor machine availability, cultural and socio-economic aspects, high-cost treatment without reimbursement, and administrative and governmental barriers [18].

## Conclusions

ICU medical staff exhibit good knowledge, a positive attitude and appropriate practices for CRRT. Males were more confident in their knowledge compared to females. Extending CRRT practice time, pursuing further study at a general ICU or a teaching hospital, joining a CRRT specialist panel, and increasing overall educational level can enhance ICU staff’s knowledge, attitude, and practice levels towards CRRT. CVVH and CVVHDF are the predominant modes in Central and South China ICUs. Department training and accumulation of work experience are primary methods for acquire CRRT-related knowledge. Considering the convenience of training programs will enhance ICU staff’s willingness to participate. Training sessions can focus on CRRT liquid management, prevention and treatment of vascular access complications, common alarm causes and handling methods, and the basic CRRT principles.

### Supplementary Information

**Supplementary Material 1.**

**Supplementary Material 2.**

## Data Availability

The datasets generated and/or analyzed during the current study are not publicly available due to the sensitive nature of the interview questions but are available from the corresponding author on reasonable request.

## Abbreviations

CRRT: Continuous renal replacement therapy
RRT: Renal replacement therapy
ICU: Intensive care unit
SCT: A specialized CRRT team
KAP: Knowledge, attitude, and practice
ANOVA: Analysis of variance
COVID-19: Coronavirus disease 2019
SPSS: Statistic package for social science
IBM: International business machine
USA: The United States of America
CVVH: Continuous venovenous hemofiltration
CVVHDF: Continuous venovenous hemodiafiltration
CVVHD: Continuous venovenous hemodialysis
SCUF: Slow continuous ultrafiltration
ADQI: Acute dialysis quality initiation
CAVH: Continuous arteriovenous hemofiltration
CAVHDF: Continuous arteriovenous hemodiafiltration
CAVHD: Continuous arteriovenous hemodialysis
WM: Working memory
NUF: Net ultrafiltration
HIRRT: Hemodynamic instability related to renal replacement therapy
IVCD: Inferior vena cava diameter
