# Supplementary material for: Knowledge, attitude, practice, needs, and implementation status of intensive care unit staff toward continuous renal replacement therapy: a survey of 66 hospitals in central and South China
Source: BMC Nurs. 2024 Apr 26;23:281. doi: 10.1186/s12912-024-01953-6 (PMC11055233; doi:10.1186/s12912-024-01953-6)
Supplement: Supplementary file 1 — Supplementary Material 1. [file 12912_2024_1953_MOESM1_ESM.docx]

**Knowledge, attitude, practice, needs, and implementation status of intensive care unit staff toward continuous renal replacement therapy: a survey of 66 hospitals in Central and South China**

(Protocol Number: 2022 csxx)

**Questionnaire**

**Site name: ________________________________**

**Respondent's signature: ________________________________**

Version Number / Date: V 1.0 / 20220911

**Questionnaire, form-filling instructions**

1. **This questionnaire must be completed by the respondent, and the applicant must fully understand the study party case and the required diagnosis and treatment information.**
2. **This questionnaire is filled in through Wechat online.**
3. **Mark them in the ○ of all selected items.**

**4. The respondent must sign the beginning and last page of the questionnaire to indicate that it has been checked**

**All the data in the questionnaire and confirmed that they was complete and accurate.**

**The arrangement standard**

| Selection criteria | | |
| --- | --- | --- |
| 1 | Registered physician and nurse in critical care medicine | whether or not   - □ |
| 2 | Working in the critical care department for more than or equal to 1 year | whether or not   - □ |
| 3 | Signed informed consent (as included in the questionnaire) | whether or not   - □ |

**Note: If any of the above 3 answers is "No", the respondent will not participate in this study**

| **Exclusion criteria** | | |
| --- | --- | --- |
| 1 | Leave during the survey | whether or not   - □ |
| 2 | Regular training students | whether or not   - □ |

**Note: If any of the above answers is "yes", the respondent will not participate in this study**

**ICU care CRRT questionnaire and assignment**

Dear critical care colleagues, in order to understand the current status of CRRT care, we specially designed this questionnaire for critical care patients. This questionnaire was completed anonymously, and it is expected to take about 5min. Thank you for your strong support and cooperation. Please combine your own real situation, there is no right or wrong option, and your answer is limited to this study. If you voluntarily fill in and submit it, you will voluntarily fill in and give informed consent. Thank you again for your support!

1. Sex [single choice] *

| ○ 1, male |
| --- |
| ○ 2, female |

2. Your age: [Multiple choice] *

| ○ 1, 20-30 years old | ○ 2, 31-40 years old | ○ 3, 41-50 years old |
| --- | --- | --- |

3. Education: [Single choice] *

| ○ 1, below bachelor | ○ 2, bachelor | ○ 3, Master or above |
| --- | --- | --- |

4. Professional title: [Single choice] *

| ○ 1, primary | ○ 2, intermediate | ○ 3, Deputy senior level and above |
| --- | --- | --- |

5. Your hospital grade [Multiple choice] *

| ○ 1, tertiary synthesis |
| --- |
| ○ 2, tertiary specialty |
| ○ 3, Grade II |
| ○ 4, others |

6. Name of your hospital [fill in the blank question] *

_________________________________

7. Area of your hospital [Single choice] *

| ○ 1, Northeast |
| --- |
| ○ 2, North China |
| ○ 3, East China |
| ○ 4, Central China |
| ○ 5, South China |
| ○ 6, Southwest |
| ○ 7, Northwest |

8. Is your hospital a teaching hospital directly under the Medical school?[single choice]*

| ○ 1, is |
| --- |
| ○ 2, no |

9. Working years: [Single choice] *

| ○ 1,1-2 years | ○ 1,3-5 years | ○ 3,6-10 years | ○ 4,10 years |
| --- | --- | --- | --- |

10. What years of CRRT treatment operation experience?[single choice]*

| ○ 1, <1 year |
| --- |
| ○ 2,1-2 years |
| ○ 3,3-5 years |
| ○ 4,6-10 years |
| ○ 5,> 10 years |

11. Your occupation [Multiple choice] *

| ○ 1, Doctor |
| --- |
| ○ 2, Nurse |

12. Whether it is an administrative officer [single choice] *

| ○ 1, is |
| --- |
| ○ 2, no |

13. Your ICU category [Multiple choice] *

| ○ 1. General |
| --- |
| ○ 2. Specialist |

14. Are you a member of the CRRT specialist team [Single choice] *

| ○ 1, is |
| --- |
| ○ 2, no |

15. CRRT patient mode in your department [Single choice] *

| ○ 1, nurse-patient (CRRT patients) ratio: 1:1 |
| --- |
| ○ 2, nurse-patient (CRRT patients) ratio: 1:2 |

16. Your way to receive CRRT education and training [Multiple choice] *

| □ 1. Academic conference lectures outside the hospital |
| --- |
| □ 2. Knowledge training in the hospital |
| □ 3. Department knowledge training |
| □ 4. Work experience |
| □ 5. Communication within the hospital |
| □ 6. Professional academic papers |
| □ 7. Web materials and lectures |

17. What you want to learn in the CRRT training [Multiple choice] *

| □ 1. CRRT basic principles |
| --- |
| □ 2. Common alarm causes and handling methods |
| □ 3. Prevention and treatment of vascular access complications |
| □ 4. CRRT liquid management |
| □ 5. Other _________________ |

18. Your current method of assessing volume in the treatment management of CRRT patients [multiple choice] *

| □ 1. Vital signs |
| --- |
| □ 2. Ultrasound |
| □ 3. Arteriovenous blood gas analysis |
| □ 4. Experience |

19. The CRRT mode you often choose is [multiple choice] *

| □1. CVVH |
| --- |
| □2. CVVHD |
| □3. CVVHDF |
| □4. SCUF |

20 Frequency of tube blockage during CRRT treatment [single choice] *

| ○ 1. Never seen it before |
| --- |
| ○ 2. Occasionally |
| ○ 3. Frequent( >3 cases in 1 month) |
| ○ 4. No attention |

21. Frequency of unplanned treatment during CRRT [single choice] *

| ○ 1. Never seen it before |
| --- |
| ○ 2. Occasionally |
| ○ 3. Frequent( >3 cases in 1 month) |
| ○ 4. No attention |

22 Frequency of blood pressure drop during CRRT [single choice] *

| ○ 1. Never seen it before |
| --- |
| ○ 2. Occasionally |
| ○ 3. Frequent( >3 cases in 1 month) |
| ○ 4. No attention |

23. Please choose the actual corresponding options according to your familiarity with the CRRT related content.[Matrix single-choice] *

|  | 1. Very unfamiliar | 2. Unfamiliar | 3. Uncertainty | 4. Familiar | 5. Very familiar |
| --- | --- | --- | --- | --- | --- |
| 1. Basic CRRT principle | ○ | ○ | ○ | ○ | ○ |
| 2. Timing of CRRT initiation | ○ | ○ | ○ | ○ | ○ |
| 3. Treatment mode selection | ○ | ○ | ○ | ○ | ○ |
| 4. Parameter setting meaning and adjustment | ○ | ○ | ○ | ○ | ○ |
| 5. Differences between different dilution modes | ○ | ○ | ○ | ○ | ○ |
| 6. Selection and adjustment of anticoagulation method | ○ | ○ | ○ | ○ | ○ |
| 7. Alarm identification and treatment | ○ | ○ | ○ | ○ | ○ |
| 8. Identification and management of complications | ○ | ○ | ○ | ○ | ○ |
| 9. Liquid and electrolyte management | ○ | ○ | ○ | ○ | ○ |
| 10. Maintenance of vascular access | ○ | ○ | ○ | ○ | ○ |
| 11. Maintenance of vascular access | ○ | ○ | ○ | ○ | ○ |
| 12. CRRT machine maintenance | ○ | ○ | ○ | ○ | ○ |
| 13. CRRT-related documents and records | ○ | ○ | ○ | ○ | ○ |
| 14. Withdrawal time of the patient for CRRT | ○ | ○ | ○ | ○ | ○ |

24. Please choose the appropriate option according to the current status of CRRT treatment.[Matrix single-choice] *

|  | 1. Highly disagree | 2. Disagree | 3. No idea | 4. Agree | 5. Highly agree |
| --- | --- | --- | --- | --- | --- |
| 1. I can manage the whole CRRT process of patients with confidence | ○ | ○ | ○ | ○ | ○ |
| 2. Pay attention to CRRT alarm and treatment | ○ | ○ | ○ | ○ | ○ |
| 3. Doctors should seek advice from nurses when prescribing CRRT | ○ | ○ | ○ | ○ | ○ |
| 4. Medical care integration mode should be implemented in CRRT management | ○ | ○ | ○ | ○ | ○ |
| 5. Unplanned interruption of CRRT Sessions should be analyzed and discussed | ○ | ○ | ○ | ○ | ○ |
| 6. It is necessary to calculate downtime (inefficiency) during CRRT | ○ | ○ | ○ | ○ | ○ |
| 7. CRRT is the best fluid management method for critically ill patients | ○ | ○ | ○ | ○ | ○ |
| 8. Medical staff who use CRRT need to pass an examination before performing CRRT | ○ | ○ | ○ | ○ | ○ |
| 9. Systematic CRRT training can improve the professional ability of medical staff | ○ | ○ | ○ | ○ | ○ |
| 10. Nurses can regulate the ultrafiltration rate independently | ○ | ○ | ○ | ○ | ○ |
| 11. When nurses find problems with CRRT treatment orders, they should provide timely feedback to doctors | ○ | ○ | ○ | ○ | ○ |
| 12. When dealing with CRRT emergencies of patients, consult CRRT specialist team members as soon as necessary | ○ | ○ | ○ | ○ | ○ |
| 13. Arteriovenous reverse connection affects the therapeutic effect of CRRT | ○ | ○ | ○ | ○ | ○ |
| 14. During CRRT, nurses can regulate the citrate infusion rate independently | ○ | ○ | ○ | ○ | ○ |
| 15. In the treatment of CRRT, transfusion of blood products can increase the risk of CRRT clotting | ○ | ○ | ○ | ○ | ○ |

25. Please select the appropriate option according to the current situation of CRRT operation.[Matrix single-choice] *

|  | 1. No | 2. Yes |
| --- | --- | --- |
| 1. Your department has established a CRRT specialist panel | ○ | ○ |
| 2. Not treating all patients with a uniform CRRT prescription (same parameters for all patients) | ○ | ○ |
| 3. Hemodynamics were evaluated during CRRT | ○ | ○ |
| 4. Therapeutic dose and filtration fraction were calculated during CRRT | ○ | ○ |
| 5. Adjust the ultrafiltration rate of the patient every hour | ○ | ○ |
| 6. Exam and adjust CRRT setting in time when clotting occurs in the CRRT circulation line | ○ | ○ |
| 7. Before the start of CRRT, doctors and nurses should make joint decisions on the formulation of anticoagulation methods and goals | ○ | ○ |
| 8. During CRRT, when the vascular access flow was poor, I chose to adjust the catheter position as soon as possible | ○ | ○ |
| 9. Medical and nursing staff should work together to solve alarms | ○ | ○ |
| 10. During CRRT, the treatment plan should be adjusted when the patients' condition | ○ | ○ |

Fill in it is over, thank you for your support!

**Table S1 presents the assignment of each variable such as demographics and work characteristics.**

**Table S1.** Variable assignment

| Variable | Assignment |
| --- | --- |
| Gender | Male = 1, Female = 2 |
| Age | 20 to 30 Years = 1, 31 to 40 Years = 2, 41 to 50 Years = 3 |
| Educational level | College or below =1  Bachelor =2  Master or above =3 |
| Professional title | Junior =1  Intermediate =2  Deputy senior or above =3 |
| Hospital grade | Tertiary Grade A general =1  Secondary specialized =2  Secondary or other =3 |
| Teaching hospital | Yes= 1, No = 2 |
| Working years | 1 to 2 years = 1, 3 to 5 years = 2, 6 to 10 years = 3, >10 years = 4 |
| Years of CRRT practice | ≤ 1 years = 1, 1 to 2 years = 2, 3 to 5 years = 3, 6 to 10 years = 4, >10 years = 5 |
| Administrative personnel | Yes= 1, No= 2 |
| ICU category | General = 1, Specialized = 2 |
| CRRT specialist panel membership | Yes= 1, No= 2 |

ICU: intensive care unit; CRRT: continuous renal replacement therapy.
