# Supplementary material for: Knowledge, attitude, practice, needs, and implementation status of intensive care unit staff toward continuous renal replacement therapy: a survey of 66 hospitals in central and South China
Source: BMC Nurs. 2024 Apr 26;23:281. doi: 10.1186/s12912-024-01953-6 (PMC11055233; doi:10.1186/s12912-024-01953-6)
Supplement: Supplementary file 2 — Supplementary Material 2. [file 12912_2024_1953_MOESM2_ESM.docx]

**Supplementary Table 1 .**Knowledge of ICU staff toward CRRT

| Knowledge Point | Knowledge(n, %) | | | | |
| --- | --- | --- | --- | --- | --- |
|  | Very unfamiliar | Unfamiliar | Uncertainty | Familiar | Very familiar |
| 1.Basic CRRT principle | 7(1.77%) | 25(6.33%) | 53(13.42%) | 260(65.82%) | 50(12.66%) |
| 2.Timing of CRRT initiation | 8(2.03%) | 12(3.04%) | 62(15.7%) | 269(68.10%) | 44(11.14%) |
| 3. Treatment mode selection | 10(2.53%) | 17(4.30%) | 66(16.71%) | 257(65.06%) | 45(11.39%) |
| 4. Parameter setting meaning and adjustment | 10(2.53%) | 22(5.57%) | 84(21.27%) | 238(60.25%) | 41(10.38%) |
| 5. Differences between different dilution modes | 14(3.54%) | 28(7.09%) | 92(23.29%) | 224(56.71%) | 37(9.37%) |
| 6. Selection and adjustment of anticoagulation method | 9(2.28%) | 18(4.56%) | 77(19.49%) | 258(65.32%) | 33(8.35%) |
| 7. Alarm identification and treatment | 7(1.77%) | 23(5.82%) | 89(22.53%) | 241(61.01%) | 35(8.86%) |
| 8. Identification and management of complications | 8(2.03%) | 24(6.08%) | 88(22.28%) | 247(62.53%) | 28(7.09%) |
| 9. Liquid and electrolyte management | 7(1.77%) | 25(6.33%) | 83(21.01%) | 250(63.29%) | 30(7.59%) |
| 10. Maintenance of vascular access | 9(2.28%) | 29(7.34%) | 72(18.23%) | 251(63.54%) | 34(8.61%) |
| 11. Pausing treatment self-circulation | 13(3.29%) | 45(11.39%) | 106(26.84%) | 204(51.65%) | 27(6.84%) |
| 12. CRRT machine maintenance | 16(4.05%) | 58(14.68%) | 116(29.37%) | 177(44.81%) | 28(7.09%) |
| 13. CRRT-related documents and records | 11(2.78%) | 28(7.09%) | 67(16.96%) | 247(62.53%) | 42(10.63%) |
| 14.Withdrawal time of the patient for CRRT | 8(2.03%) | 19(4.81%) | 87(22.03%) | 251(63.54%) | 30(7.59%) |

CRRT: continuous renal replacement therapy.

**Supplementary Table 2.**Attitude of ICU staff toward CRRT

| Viewpoint | Attitudes(n, %) | | | | |
| --- | --- | --- | --- | --- | --- |
|  | Highly disagree | Disagree | No idea | Agree | Highly agree |
| 1. I can manage the whole CRRT process of patients with confidence | 6(1.52%) | 11(2.78%) | 96(24.3%) | 238(60.25%) | 44(11.14%) |
| 1. Pay attention to CRRT alarm and treatment | 2(0.51%) | 2(0.51%) | 34(8.61%) | 271(68.61%) | 86(21.77%) |
| 3.Doctors should seek advice from nurses when prescribing CRRT | 2(0.51%) | 32(8.10%) | 97(24.56%) | 214(54.18%) | 50(12.66%) |
| 4.Medical care integration mode should be implemented in CRRT management | 0(0%) | 1(0.25%) | 22(5.57%) | 232(58.73%) | 140(35.44%) |
| 5.Unplanned interruption of CRRT Sessions should be analyzed and discussed | 0(0%) | 2(0.51%) | 34(8.61%) | 236(59.75%) | 123(31.14%) |
| 6.It is necessary to calculate downtime (inefficiency) during CRRT | 0(0%) | 5(1.27%) | 53(13.42%) | 256(64.81%) | 81(20.51%) |
| 7.CRRT is the best fluid management method for critically ill patients | 2(0.51%) | 18(4.56%) | 90(22.78%) | 221(55.95%) | 64(16.2%) |
| 8.Medical staff who use CRRT need to pass an examination before performing CRRT | 0(0%) | 2(0.51%) | 34(8.61%) | 259(65.57%) | 100(25.32%) |
| 9.Systematic CRRT training can improve the professional ability of medical staff | 0(0%) | 3(0.76%) | 19(4.81%) | 240(60.76%) | 133(33.67%) |
| 10.Nurses can regulate the ultrafiltration rate independently | 12(3.04%) | 80(20.25%) | 122(30.89%) | 156(39.49%) | 25(6.33%) |
| 11.When nurses find problems with CRRT treatment orders, they should provide timely feedback to doctors | 0(0%) | 1(0.25%) | 16(4.05%) | 236(59.75%) | 142(35.95%) |
| 12.When dealing with CRRT emergencies of patients, consult CRRT specialist team members as soon as necessary | 1(0.25%) | 5(1.27%) | 34(8.61%) | 250(63.29%) | 105(26.58%) |
| 13.Arteriovenous reverse connection affects the therapeutic effect of CRRT | 3(0.76%) | 18(4.56%) | 79(20.00%) | 216(54.68%) | 79(20.00%) |
| 14. During CRRT, nurses can regulate the citrate infusion rate independently | 23(5.82%) | 101(25.57%) | 112(28.35%) | 126(31.90%) | 33(8.35%) |
| 15.In the treatment of CRRT, transfusion of blood products can increase the risk of CRRT clotting | 2(0.51%) | 39(9.87%) | 113(28.61%) | 202(51.14%) | 39(9.87%) |

ICU: intensive care unit; CRRT: continuous renal replacement therapy.

**Supplementary Table 3.**Practice of ICU staff toward CRRT

| Behavior | Yes | No |
| --- | --- | --- |
| 1.Your department has established a CRRT specialist panel | 280(70.89%) | 115(29.11%) |
| 2.Not treating all patients with a uniform CRRT prescription (same parameters for all patients) | 268(67.85%) | 127(32.15%) |
| 3.Hemodynamics were evaluated during CRRT | 371(93.92%) | 24(6.08%) |
| 4.Therapeutic dose and filtration fraction were calculated during CRRT | 323(81.77%) | 72(18.23%) |
| 5.Adjust the ultrafiltration rate of the patient every hour | 285(72.15%) | 285(72.15%) |
| 6.Exam and adjust CRRT setting in time when clotting occurs in the CRRT circulation line | 285(72.15%) | 285(72.15%) |
| 7. Before the start of CRRT, doctors and nurses should make joint decisions on the formulation of anticoagulation methods and goals | 285(72.15%) | 285(72.15%) |
| 8. During CRRT, when the catheter flow was poor, I chose to adjust the catheter position as soon as possible | 285(72.15%) | 285(72.15%) |
| 9. Medical and nursing staff should work together to solve alarms | 285(72.15%) | 285(72.15%) |
| 10. During CRRT, the treatment plan should be adjusted when the patient's status changes | 285(72.15%) | 285(72.15%) |

CRRT: continuous renal replacement therapy.

**Supplementary Table 4.**Correlation of knowledge, attitude and practice level of ICU staff toward CRRT (n = 395).

| Level | r | p value |
| --- | --- | --- |
| Knowledge-attitude | 0.431 | ＜0.001 |
| Knowledge-practice | 0.250 | ＜0.001 |
| Attitude-practice | 0.176 | ＜0.001 |

ICU: intensive care unit; CRRT: continuous renal replacement therapy.

r : Pearson correlation coefficient.

p value <0.05 was considered as a significant difference.
